# Immunological and Clinical Impact of Manipulated and Unmanipulated DLI after Allogeneic Stem Cell Transplantation of AML Patients

**DOI:** 10.3390/jcm9010039

**Published:** 2019-12-23

**Authors:** Jochen Greiner, Marlies Götz, Donald Bunjes, Susanne Hofmann, Verena Wais

**Affiliations:** 1 Department of Internal Medicine, Diakonie Hospital Stuttgart, 70176 Stuttgart, Germany; 2 Department of Internal Medicine III, University of Ulm, 89081 Ulm, Germany; marlies.goetz@uni-ulm.de (M.G.); donald.bunjes@uniklinik-ulm.de (D.B.); Verena.Wais@uniklinik-ulm.de (V.W.); 3 Department of Internal Medicine V, University of Heidelberg, 69120 Heidelberg, Germany; susanne.hofmann@med.uni-heidelberg.de

**Keywords:** allogeneic stem cell transplantation (allo-SCT), donor lymphocyte infusion (DLI), graft-versus-leukemia (GvL) effect, relapse, virus-specific T cells, α/β T depletion

## Abstract

Allogeneic stem cell transplantation (allo-SCT) is the preferred curative treatment for several hematological malignancies. The efficacy of allo-SCT depends on the graft-versus-leukemia (GvL) effect. However, the prognosis of patients with relapsed acute myeloid leukemia (AML) following allo-SCT is poor. Donor lymphocyte infusion (DLI) is utilized after allo-SCT in this setting to prevent relapse, to prolong progression free survival, to establish full donor chimerism and to restore the GvL effect in patients with hematological malignancies. Thus, there are different options for the administration of DLI in AML patients. DLI is currently used prophylactically and in the setting of an overt relapse. In addition, in the minimal residual disease (MRD) setting, DLI may be a possibility to improve overall survival. However, DLI might increase the risk of severe life-threatening complications such as graft-versus-host disease (GvHD) as well as severe infections. The transfusion of lymphocytes has been tested not only for the treatment of hematological malignancies but also chronic infections. In this context, manipulated DLI in a prophylactic or therapeutic approach are an option, e.g., virus-specific DLI using different selection methods or antigen-specific DLI such as peptide-specific CD8+ cytotoxic T lymphocytes (CTLs). In addition, T cells are also genetically engineered, using both chimeric antigen receptor (CAR) genetically modified T cells and T cell receptor (TCR) genetically modified T cells. T cell therapies in general have the potential to enhance antitumor immunity, augment vaccine efficacy, and limit graft-versus-host disease after allo-SCT. The focus of this review is to discuss the different strategies to use donor lymphocytes after allo-SCT. Our objective is to give an insight into the functional effects of DLI on immunogenic antigen recognition for a better understanding of the mechanisms of DLI. To ultimately increase the GvL potency without raising the risk of GvHD at the same time.

## 1. Introduction

Donor lymphocyte infusion (DLI) holds curative potential for acute myeloid leukemia (AML) patients due to the augmentation of the graft-versus-leukemia (GvL) effect. However, DLI may cause graft-versus-host disease (GvHD), which could become life threatening. A better understanding of specific T cell responses against leukemic cells could escalate GvL potency without increasing the risk of GvHD.

**Figure 1:**
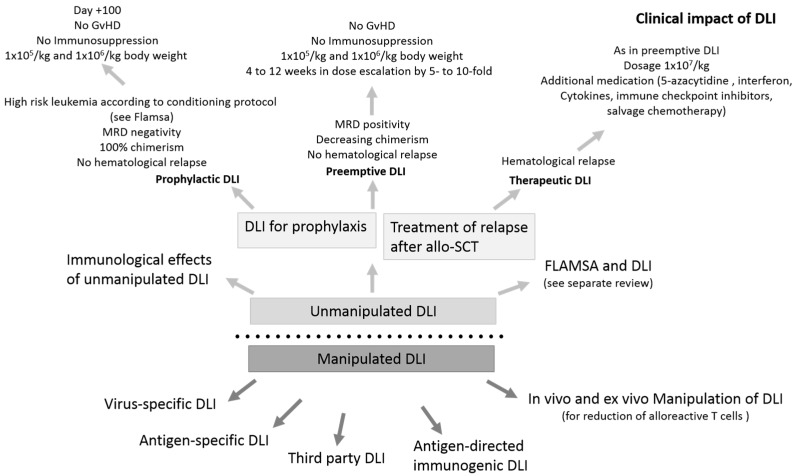
Different modalities and conditions of unmanipulated and manipulated donor lymphocyte infusion (DLI). Unmanipulated DLI is administered to prevent relapse in a prophylactic situation as well as to treat relapse. This has an immunological and, in the majority of cases, also a clinical impact. As to manipulated DLI, this manipulation may take place in vivo or ex vivo. GvHD, graft-versus-host disease; MRD, minimal residual disease; allo-SCT, Allogeneic stem cell transplantation.

DLI has been in use for approximately 30 years as a kind of adoptive T cell therapy and was first administered in chronic myeloid leukemia (CML) patients suffering from relapse after allogeneic stem cell transplantation (allo-SCT) [1]. DLI is a potent approach to enable remission after relapse, despite the risk of inducing GvHD (Figure 1). The best response rates were achieved in CML (80%), while in other hematological diseases, it was less effective [2]. Delayed DLI as a T cell boost as well as gradual dose escalation with repeated DLI is utilized to date in clinical practice to reduce GvHD risk [3]. Some technologies are under development and could play a significant role in the future. It has become clear that DLI can be administered at a much later time point. This allows for the manipulation of the T cell response, such as the selective depletion of alloreactive T cells or the introduction of molecular kill switches, which enable the termination of T cell activity in severe GvHD. These technologies could be particularly important in a haploid setting.

Additional new immunotherapeutic T cell approaches are genetically modified affinity enhanced T cell receptor (TCR) against several leukemia-associated antigens (LAAs). LAAs such as New York esophageal squamous cell carcinoma-1 (NYESO-1) in multiple myeloma [4] or Wilms tumor antigen 1 (WT1)-TCR in AML and myelodysplastic syndrome (MDS) [5,6,7] have been clinically investigated and can be employed both in a autologous and in a allogeneic setting. Some were tested in clinical phase I/II peptide vaccination trials and showed immunological as well as clinical responses. Another approach includes T cell receptor (TCR)-gene modified T cells as well as chimeric antigen receptor (*CAR*) gene-modified T cells [8,9].

Many of these approaches may be applied to selected patients in the future, but there are several challenges. The best combinations and targets with high GvL potency and reduced GvHD risk have to be selected and the ideal cytokine milieu for therapy as well as the right T cell composition have to be discovered (Table 1).

A better comprehension of the interaction between DLI and the corresponding targets might facilitate the increase in GvL potency, without escalating the GvHD risk, which is of utmost importance.

## 2. Understanding the Functionality of DLI—Immunological Effects of Unmanipulated DLI

Although unmanipulated DLI is commonly used in the clinical setting, the immunological mechanisms have to be further elucidated. In particular, the role of antigen-specific T cell responses after DLI and a more extensive comprehension of leukemia elimination by T cells is mandatory in established immunotherapies such as allo-SCT and DLI. The main goal is to increase the GvL potency without raising the risk of GvHD at the same time. The most applied technique is the infusion of unmanipulated DLI after unmanipulated or in vivo T cell depleted transplantation from matched sibling or unrelated donors in patients with AML or MDS. After allo-SCT, tissue damage is gradually repaired. In this process, donor dendritic cells (DCs) replace the recipient DCs within the first 6 months after allo-SCT. Accordingly, the host and donor immune subsets do progressively adapt. This explains the clinical observation that a higher number of T cells can be administered without induction of severe GvHD (less than 10^5^/kg body weight after 3 months, to 10^6^/kg body weight at 6 months) [10]. Therefore, DLI should only be administered in the absence of tissue damage and inflammatory circumstances, for example without GvHD and uncontrolled infections. To date, these infusions are not guided by the diversity of the TCR repertoire or the subsets of lymphocytes [11,12].

For a better comprehension of the immunological function of DLI and for the recognition of immunogenic leukemia-associated antigens (LAAs), Hofmann et al. [13] assessed the frequency and diversity of LAA-specific cytotoxic T cells in a small patient cohort, before and after DLI. Patients were screened for LAA-specific cytotoxic T lymphocyte (CTL) responses, number of Tregs and cytokine levels before and after DLI. Several LAAs—among them, preferentially expressed antigen in melanoma (PRAME), WT1, receptor for hyaluronan acid-mediated motility (RHAMM), and NYESO-1—were tested for specific CTL responses before and after DLI. A significant increase in the number of LAAs recognized by CTLs in clinical responders after DLI and an enhanced LAA diversity in T cell responses were detected. Thus, clinical responses after allo-SCT and DLI might be dependent on an increase in the frequency and diversity of LAA-specific T cell responses. The assumption is that several LAAs play a role in CTL response after DLI and the increase in CTL specific LAA-detection is especially decisive for a successful clinical response to DLI. The diversity of antigen-specific T cells seems to have a strong influence on the GvL effect after allo-SCT and DLI. The conjunction of all these factors may contribute to the clinical outcome of patients treated with several DLI applications.

Moreover, clinical responders showed a significant decrease in the frequency of the highly immunosuppressive CD4+ Tregs. The quantity of Tregs remained stable in non-responders [13]. Tregs play a central role in the maintenance of self-tolerance and promote malignant cell progression by suppressing effective antitumor immunity [14], and thus it is truly striking that clinical responders in the analyzed patient cohort show a significant reduction of Treg. Further studies detected an association with a higher frequency of Treg and unfavorable clinical outcome in several other tumor entities including hematological malignancies [15,16,17,18,19].

These data imply that DLI may not only qualify for mono-therapeutic use but also for combined approaches. Thus, the reduction of Treg could improve the efficacy of other immunotherapies or immune checkpoint inhibitors [20].

In another analysis, patients with Nucleophosmin 1 (NPM1)-mutated AML were treated with DLI after allo-SCT. The authors detected immune responses against different LAAs, especially against NPM1-derived epitopes of the mutated region of NPM1. The detection of the immune responses was linked to minimal residual disease (MRD) negativity, therefore suggesting a correlation of GvL and LAA-specific CTL response [21]. Interestingly, in a cohort of 25 patients with NPM1-mutated AML, the presence of CTL responses against the immunogenic region of NPM1 was associated with a longer overall survival [22]. Due to a lack of tolerance against mutant-derived neoantigen epitopes, these are promising targets for immunotherapy and are currently particularly in the focus for checkpoint inhibitor therapies. Moreover, a correlation of mutational antigen load and clinical benefit was described for melanoma and non-small-cellular lung cancer [23,24]. Neoantigens derived from the mutated region of NPM1 are interesting targets in AML. 

The cytokine milieu may influence the function of DLI. Moore et al. [25] show that interleukin-7 (IL-7) and IL-2 are homeostatic cytokines for naive CD4+ and CD8+ T cells. Furthermore, in high concentrations, IL-15 provides a setting for the directed expansion of in vitro-derived memory/effector CD8+ T cell populations that have been adoptively transferred. Yet, IL-15 has to be further tested in phase I trials.

Based on this concept, cytokine-induced killer (CIK) cells have been developed. CIK cells are memory T lymphocytes, which have acquired CD56 expression. In several experimental allogeneic models, CIK cells have demonstrated in vitro and in vivo antitumor activity, direct intratumor homing following intravenous administration and, more importantly, reduced GvHD activity. Finally, the study suggests that CIK cells may be effective in the treatment of post-transplant relapse [26]. Further scientific studies are necessary to improve the understanding of DLI and to increase the efficacy of DLI and DLI in combination with other drugs.

## 3. Clinical Impact of Unmanipulated DLI in AML

### 3.1. Therapeutic DLI for the Treatment of Morphological Relapse

Therapeutic DLI is well established in the treatment of clinical relapse in different hematological malignancies. The response rate and survival after DLI vary from entity to entity and depend on several factors, such as disease characteristics and the genotype of the disease, disease burden, the proliferative rate of the disease, donor origin, as well as the clinical situation of the patient. 

In 1997, Collins et al. [27] published a retrospective study with 140 patients in 25 North American programs with relapsed malignancies (CML, AML, ALL, MDS and myeloma) after allo-SCT. In this study, a high percentage of patients with relapsed chronic-phase CML, DLI administration resulted in complete remission. While complete remission was observed less frequently in patients with advanced CML and acute leukemia [27]. Similar results were oberserved by Posthuma et al. [28], where DLI resulted in complete cytogenetic remission (CCR) of relapsed chronic-phase chronic myeloid leukemia (CML-CP) after allo-SCT in up to 80% of patients.

With GvHD as the main complication, in 1998, Verdonck et al. [29] evaluated the efficacy and toxicity of different doses of donor T cells. T cell doses varied from 0.1 × 10^7^ to 33 × 10^7^ T cells/kg body weight. They observed that higher T cell doses (> or = 10 × 10^7^/kg) induced serious GvHD as well as marrow aplasia [29].

Based on this, Posthuma et al. [28] reduced the dosage of DLI in CML patients, which was associated with less GvHD but also with a longer interval between treatment and CCR. Posthuma also observed that DLI resulted in complete cytogenetic remission (CCR) of relapsed chronic-phase chronic myeloid leukemia (CML-CP) after allo-SCT in up to 80% of patients. Because of the longer interval between treatment and CCR, they postulated that combining alpha-interferon (alpha-IFN) with DLI would make it feasible to decrease the dose of DLI, thereby limiting GvHD, and at the same time decrease the interval between DLI and CCR for patients with either a hematologic or cytogenetic relapse. This concept is still used today in patients with AML as well as in other hematological malignancies. Further generated methods include chemotherapy, immunosuppressive medications, and the use of selected T cell subsets and/or modified T cells (for instance, suicide gene insertion) [26,30,31,32]. In addition, composition approaches have been used to try to improve the outcome of the treatment with DLI. There are strategies to combine DLI with other drugs that stimulate the immune system and T cells such as interferon derivates, cytokines or immune checkpoint inhibitors as well as combinations of DLI with other drugs such as hypomethylating substances and other immunomodulatory agents [30,33,34,35].

To evaluate the role of DLI in the treatment of relapsed AML in comparison to further strategies, Schmid et al. analyzed 399 patients retrospectively. In total, 177 patients were treated with DLI and 228 were the controls. The survival rate two years after allo-SCT was 21% for patients receiving DLI and 9% for patients without DLI treatment (*p* = 0.04). Among DLI recipients, a lower tumor burden at relapse (<35% of bone marrow blasts; *p* = 0.006) and favorable cytogenetics (*p* = 0.004) were predictive for survival in a multivariate analysis. Two-year survival was 15% ± 3% if DLI was administered in aplasia or in active disease [36].

The European Society for Blood and Marrow Transplantation (EBMT) Acute Leukemia Working Group conducted a retrospective study of AML patients in complete remission (CR) and relapse after allo-SCT. In 32%, CR could be reinduced, but long-term survival was almost exclusively achieved after successful induction of CR by cytoreductive therapy, followed either by DLI or by a second allo-SCT [37].

Retrospective studies found the combination of Sorafenib with DLI in FLT3-ITD+ AML with relapse after allo-SCT to be superior to treatment with DLI alone [38,39]. De Freitsas et al. retrospectively collected data of Sorafenib, partially in combination with hypomethylating agents and DLI. Hematological response was documented in 12 of 13 patients (92%), and five of 13 (38%) achieved CR. GvHD was frequently observed in association with DLI. Therefore, Sorafenib might represent a valid treatment option; however, prospective and larger studies are needed [40].

In particular, the combination of DLI with hypomethylating agents seems to be a very effective therapy for relapsed MDS and AML patients after allo-SCT [41,42,43]. In a phase I study [43], a phase II study [42] and several retrospective analyses [44,45,46], this was shown. A relevant number of the patients included showed significantly improved survival rates with acceptable toxicity [41,42,43]. For example, in a retrospective study with azacytidine and DLI, the overall response rate was 33% and the 2 year overall survival (OS) was 29% [45]. Nonetheless, it has to be considered that molecular relapse alone, diagnosis of MDS and low marrow blast count at the time of relapse are associated with better OS [38]. In a retrospective study, treatment with decitabine and DLI as alternative substance showed a response rate of 25%, including patients with previous azacytidine failure, and a 2 year OS of 11% [42]. There was no significant incidence of acute GvHD (aGvHD) or chronic GvHD (cGvHD). According to these data, hypomethylating agents in combination with DLI may be considered in patients who might not be eligible for a more aggressive remission induction [38]. For long-term disease control after relapse, a second allo-SCT has to be considered [38]. Patients with an MDS relapse or AML with low disease burden after allo-SCT seem to benefit more from azacytidine and DLI therapy, than patients with AML [45]. There are currently no specific data on these aspects.

If possible, in the case of bulky and fast-growing disease, intensive chemotherapy should be chosen rather than hypomethylating agents, as in a retrospective analysis, chemotherapy was superior, considering OS [47]. 

Especially in cases of high tumor burden, conventional chemotherapy should be considered. However, chemotherapy alone generally has no curative potential in this setting. To overcome the reduced effectiveness of DLI in these circumstances, Levine et al. used a chemotherapy strategy to debulk disease before administration of DLI. 65 patients were prospectively treated with cytarabine-based chemotherapy, followed by DLI. In total, 27 of 57 assessable patients achieved CR. GvHD was observed in 56% of the patients. Overall survival at 2 years for the entire cohort was 19%. Patients in CR were more likely to survive, with 1 and 2 year survival rates of 51% and 41%, respectively. In conclusion, treatment with chemotherapy before DLI can help patients with advanced myeloid relapse. However, patients with short remissions after allo-SCT are unlikely to benefit from this approach [48].

The possibility of combining DLI with chemotherapy was also evaluated in several other studies [49]. In the combination therapy, DLI is administered either at the time of the leukocyte nadir or after regeneration. DLI administration in the leukocyte nadir does not require sustained response but has a higher risk of toxicity. DLI after regeneration could reduce the GvHD risk but might not be appropriate in some patients without sustained response. Furthermore, in a retrospective study, it was demonstrated that intensive chemotherapy administered with a second allo-SCT or DLI is superior to chemotherapy alone in relapsed MDS after allo-SCT; OS was 32% in the immunotherapy group, 6% in the cyto-reductive chemotherapy only group, and 2% in the palliative care-only group (*p* < 0.001) [49]. Another option is chemotherapy followed by DLI and azacytidine, and for further insight, a phase I study was conducted in patients with AML relapse [43]. Nonetheless, prospective studies are needed [38].

Another concept for the treatment of AML relapse after allo-SCT is the initiation of epigenetic therapy, interspersed with low dose DLI. Therefore, a phase I/II feasibility study of panobinostat alone and the combination of panobinostat and decitabine prior to DLI in patients after allo-SCT with poor and very poor-risk AML was developed (Hovon 116-trial). This trial contained three dose levels consisting of either panobinostat (PNB) (20 mg at days 1, 4, 8, 11 of a 4 week-cycle) or PNB combined with decitabine (DCB, 10 or 20 mg/m^2^ at days 1–3 of every 4 week-cycle). DLI consisted of 1 × 10^6^ CD3 T cells/kg body weight at day 90 and 3 × 10^6^ at day 180 in case of a matched sibling (sib) donor or of a 70% reduced dose in case of a matched unrelated donor (MUD). In the interim analysis, 54 patients were transplanted, and median follow up was 9 months (range: 2–25) after transplantation. In total, 41 of 54 patients received PNB alone, 13 PNB/DCB (20 mg/m^2^), and 15 PNB/DCB (10 mg/m^2^). Combining PNB with DCB at a dose of 20 mg/m^2^ was not feasible due to resulting cytopenia. OS at 12 months from transplantation was 81% (±7). Five patients died due to non-relapse mortality and five died due to relapse. Relapse-free survival (RSF) at 12 months was 66% (±9). A historical HOVON control group of very poor-risk AML CR1 recipients of allo-SCT showed an OS of 52% ± 6 at 12 months and RFS of 43% ± 5. DLI could be administered in 34 patients, including 19 receiving two DLI, and nine patients three DLI. Out of 34 recipients of DLI, severe cGvHD occurred in five (15%) patients. Collectively, these results suggest an encouraging outcome with respect to relapse and OS in patients receiving PBN alone or PBN combined with DCB followed by DLI. An international prospective randomized study is in the pipeline [50].

At present, there are no valid data for treatment with chimeric antigen receptor (*CAR*) T cells for MDS and AML, but trials are ongoing.

In conclusion, therapeutic DLI is effective in AML/MDS and is currently used with or without other agents depending on the individual disease burden and GvHD risk (Table 1) [38]. Furthermore, the presented data suggest that chemotherapy is recommended in AML/MDS relapse after allo-SCT for patients who most likely tolerate the toxicity and are eligible for subsequent treatment with either DLI or second allo-SCT [38].

### 3.2. Biology of Therapeutic DLI

High tumor burden, proliferative rate and relapse, predominantly caused by immune escape mechanisms, limit the efficacy of DLI [33,51,52]. In particular, NK cells provide acute control over leukemic activity. However, by tolerance induction over time, NK cells lose their antileukemic reaction [53]. Other subsets such as gamma/delta (γ/δ) T cells appear to have a prolonged anti-leukemic effect [54]. 

In addition to the timing, frequency, setting and combination of DLI with other substances, there is still an ongoing discussion about the dosage of DLI. 

Donor type and setting, as well as the frequency and interval between infusions of DLI, have an influence on the adequate dose of DLI. Considering these factors, the recommended range in literature is 0.001 × 10^8^ to 8.8 × 10^8^ CD3+-cells/kg body weight [55]. An approach with a smaller dosage for example of 0.1–1 × 10^6^ CD3+/kg body weight, in the prophylactic setting seems reasonable. The infusion is to be repeated every four to eight weeks with an increase in the dosage by half a log level, e.g., 1. DLI: 1 × 10^6^ CD3+/kg body weight, 2. DLI: 5 × 10^6^ CD3+/kg body weight, 3. DLI: 1 × 10^7^ CD3+/kg body weight, 4. DLI: 5 × 10^7^ CD3+/kg body weight, etc. After every DLI administration, the incidence of GvHD and remission status have to be evaluated to reduce the risk of treatment-related mortality [56]. In case of preemptive or therapeutic DLI, the application of a higher starting dose is possible (5–10 × 10^6^ CD3+/kg body weight). However, the associated higher risk of GvHD has to be kept in mind [55]. 

Another aspect in this setting is the origin of DLI. Normally, DLI is collected from naïve donors as steady state lymphocytes. When donor lymphocytes are collected during stem cell apheresis, donors are pre-treated with granulocyte colony stimulating factor (G-CSF). However, the impact of G-CSF stimulation and the resulting composition of DLI on beneficial anti-leukemic responses and survival remains elusive. To evaluate the role of G-CSF-DLI, a retrospective analysis was conducted. The G-CSF-DLI patient cohort showed an improved conversion to full donor chimerism and a lower cumulative incidence of relapse or disease progression without a significantly increased cumulative incidence of GvHD [57]. DLI were examined by flow cytometry as to their cellular components. The results showed that infusion with a lower dose of CD14+ cells (<0.33 × 10^8^/kg body weight) was an independent risk factor for the occurrence of II–IV aGvHD (HR = 0.104, *p* = 0.032) in human leukocyte antigen (HLA)-identical transplant patients. In addition, a dose of CD14+ cells greater 0.33 × 10^8^/kg body weight was associated with a lower incidence of hematological relapse and longer disease-free survival (DFS) (relapse: HR = 0.193, *p* = 0.007; DFS: HR = 0.259, *p* = 0.016). However, a greater number of CD14+ cells was an independent risk factor for II–IV aGvHD (HR = 1.758, *p* = 0.034) in haploidentical allo-SCT. These data show that the cell composition of DLI provides a novel approach for the development of cellular therapies by manipulating the components of infused cells [58].

Another interesting concept first identified by Vago et al. is the potential for leukemic cells to escape immunosurveillance through loss of the mismatched HLA [59]. Therefore, uniparental HLA would escape the immunotherapeutic effect of DLI. Currently, we do not routinely monitor for HLA loss in recurrent disease. However, this would potentially allow for more targeted utilization of DLI and possibly improve the efficacy [60]. 

Overall, it seems that DLI in the preemptive setting achieves a better response than DLI administered in case of dynamic relapse [61]. Yet, DLI alone may not be the preferred strategy for treatment of manifest relapse. Repetitive DLI can be considered based, e.g., on MRD positivity, 6–8 weeks after DLI administration. The cell doses used in this setting are usually one order of magnitude higher than in a prophylactic or preemptive situation (1 × 10^7^/kg body weight) [51]. The main complication of DLI is GvHD.

### 3.3. Prophylactic Use of DLI in AML/MDS

Relapse is the most common cause of allo-SCT failure in AML. Accordingly, DLI has been routinely used in complete hematological remission without any sign of underlying disease, with full chimerism, for relapse prevention. The use of DLI in this setting is prophylactic. DLI application should be considered based on the expected risk of relapse and GvHD [38]. Generally, prophylactic DLI is administered at approximately day 100 after allo-SCT, if the patient is not under immunosuppression, and without signs of GvHD or infections.

In some studies, immunosuppressive drugs are applied concurrently with DLI [62]. However, there are discrepancies in the different results and therefore further trials are needed [38]. In case of preemptive and prophylactic use, the CD3+ dosage for the first infusion varies between 1 × 10^5^/kg and 1 × 10^6^/kg body weight and is dependent on donor type and timing [38]. In the absence of GvHD, most centers administer prophylactic DLI as a single-shot intervention, but also repetitive DLI [12,56], every 4 to 12 weeks in a dose escalation by 5- to 10-fold based on response, is feasible [38]. 

Jedlickova et al. analyzed DLI administration in high-risk AML and MDS (46 patients) at day 120 post allo-SCT with a matched control group (34 patients) in a retrospective study [12]. The OS in the DLI group compared with the control group was significantly better (7 year OS, 67% versus 31% (*p* < 0.001)). Ten patients (22%) relapsed in spite of DLI, compared with 53% in the control group. However, non-relapse mortality was low; GvHD was the main complication in the DLI group. Finally, 31/46 DLI recipients were alive and in CR at a median of 5.7 years after the first DLI. 

Schmid et al. [63] described the evaluation of efficacy of prophylactic DLI in AML patients in a registry based matched-pair analysis. Patients received DLI in complete remission and controls were matched for parameters such as age, cytogenetics, diagnosis, stage, donor, gender, conditioning and T cell depletion therapy. In total, 89 matched pairs were used for further analysis. There was no difference in survival across the entire cohort, but, notably, the authors reported significantly improved overall survival in patients with high-risk AML. Thus, prophylactic DLI is effective and may contribute to improved outcome in high-risk AML patients [63].

Furthermore, in transplantation strategies using haploidentical donors, prophylactic DLI appear to be an option to prevent relapse with an acceptable risk of GvHD and GvHD-related mortality in hematological malignancies [64,65]. Several other colleagues recorded improved outcome after prophylactic DLI [12,63,66,67]. Therefore, prophylactic DLI seems to be an effective option to prevent relapse after allogeneic stem cell transplantation and the possibilities need to be further explored in clinical phase II and III studies.

#### FLAMSA-RIC and DLI

Leukemia relapse is a major obstacle in refractory leukemia undergoing allo-SCT. To improve outcome in this cohort, a sequential intensified conditioning (fludarabine 30 mg/m^2^, high-dose cytarabine 2 g/m^2^, and amsacrine 100 mg/m^2^ from days −12 to −9 (FLAMSA-RIC)) and early rapid immunosuppressant withdrawal was invented. At this point, we will only provide a brief summary, for more information please see separate review in this special issue.

The outcome in this special risk group treated according the FLAMSA-RIC protocol is promising. The 5 year overall survival (OS) and 3 year relapse rate was 44.6% and 33.3%, respectively. To reduce the relapse risk further, prophylactic DLI was administered. Xuan and colleagues analyzed 153 refractory AML patients in a prospective study. Comparing the two groups (80 DLI versus 64 non-DLI), the relapse rate was less and OS was superior in patients receiving DLI than in those without DLI administration (22.7% vs. 33.9%, *p* = 0.048; 58.1% vs. 54.9%, *p* = 0.043). In a multivariate analysis, DLI and cGvHD were associated with less relapse and improved OS [68].

Another prospective study with FLAMSA-RIC DLI was conducted by Michallet et al. [69] in high-risk AML patients. At day +120 or 30 days after discontinuation of immunosuppressive therapy, patients received three increasing doses of donor DLI. There had to be no signs of GvHD or infections. The starting DLI dose was 1 × 10^6^ CD3+ cells/kg body weight. In total, 66 AML patients were included with a median age of 52 years. In total, 17 patients developed cGvHD (10 limited and seven extensive), five of them after DLI, with a cumulative incidence of 48% at 2 years. Patients in CR at allo-SCT benefited most from sequential intensified conditioning followed by DLI. However high rates of deadly infections were observed; therefore, the authors recommend a prophylactic anti-infectious strategy [69].

### 3.4. Preemptive Use of DLI in AML/MDS

Preemptive DLI is administered in case of persistent MRD or at the first signs of relapse, such as MRD positivity or a decreasing donor chimerism. As in the prophylactic setting, there should be no signs of GvHD. Immunosuppressant drugs preferably should already have been tapered. The dosage for DLI can be chosen slightly higher than for prophylactic DLI, according to GvHD risk and donor type (1 × 10^5^/kg and 1 × 10^6^/kg body weight), followed by repetitive DLI administration in intervals of 4–12 weeks at an escalated dose schedule and increasing the cell doses by 5- to 10-fold with each infusion, if necessary. The timing of administration depends on reappearance of MRD or mixed chimerism. So far, there is the discussion whether DLI dosage needs to be adjusted in the setting of an unrelated, related or haploidentical donor. In this context, various retrospective studies have demonstrated the effificacy of preemptive DLI [56].

In a prospective analysis, 105 patients with standard-risk acute leukemia (AML, ALL or MDS) were MRD positive after allo-SCT—of which, 49 received low-dose IL-2 only, and 56 modified DLI, with or without low-dose IL-2. The cumulative risk of relapse was significantly lower and DFS was significantly higher in patients who received DLI compared to patients who were treated only with IL-2 (*p* = 0.001 and *p* = 0.002, respectively; 3-J-OS: DLI: 58%, IL-2: 28%). These data suggest that DLI administration in patients with standard-risk acute leukemia who are MRD positive after transplantation may improve transplantation outcomes [70]. Preemptive treatment with azacytidine in MDS and AML after allo-SCT is another aspect that needs to be evaluated. The up regulation of immune signaling in cancer through the viral defense pathway is the rationale behind the combination therapy of DLI and demethylation substances, such as the DNA methyltransferase inhibitors azacytidine und decitabine [71]. In a prospective phase II study, patients with a decrease in CD34+ donor chimerism to <80%, >100 days after allo-SCT received four azacytidine cycles (75 mg/m^2^/day for 7 days) during complete hematologic remission. In total, 16 patients (80%) responded with either increasing CD34+ donor chimerism up to >80% (*n* = 10; 50%) or stabilization (*n* = 6; 30%) with the absence of relapse. Eventually, hematologic relapse occurred in 13 patients (65%), but was delayed until a median of 231 days (range, 56–558) after initial decrease in CD34+ donor chimerism to <80% [72].

Another prospective phase II study showed the efficacy of 5-azacytidine as well as 5-azacytidine in combination with DLI in patients with decreased chimerism or increasing MRD [73]. Based on these data, 5-azacytidine may be considered in patients with AML or MDS and decreasing donor chimerism [38]. Another approach in AML and MDS is DLI combined with maintenance therapies, using manipulated DLI to enhance the GvL effificacy while reducing the risk of GvHD [38]. In conclusion, pre-emptive azacytidine treatment can substantially prevent or delay hematologic relapse in patients with MDS or AML and MRD positivity after allo-SCT. Furthermore, a combination with DLI is possible.

The possibility of preemptive chemotherapy in combination with DLI application in patients with MDS and AML (*n* = 101) was analyzed in another study. The 3 year cumulative incidences of relapse, non-relapse mortality, and DFS after allo-SCT were 39.5%, 9.6%, and 51.7%, respectively. One month after Chemo-DLI 44 patients became MRD negative; their cumulative incidences of relapse and DFS significantly improved compared to those with persistent MRD one month after preemptive Chemo-DLI (relapse: 19.8% vs. 46.8%, *p* = 0.001; DFS: 69.6% vs. 46.4%, *p* = 0.004). Early onset MRD, persistent MRD after Chemo-DLI, and non-cGvHD after Chemo-DLI were associated with increased relapse and impaired DFS [74].

### 3.5. Biology of Preemptive/Prophylactic DLI

Nonetheless, it is still a challenge to separate GvL from GvHD and to find ways to enhance the GvL effect without inducing GvHD. Efforts have been made to reduce GvHD-associated morbidity and mortality by in vivo T cell depletion. This resulted in an impaired immune reconstitution, which lead to an increased incidence of opportunistic infections and a decreased GvL effect. The International Bone Marrow Transplant Registry (IBMTR) described in a retrospective study an increased leukemia relapse rate when the stem cell transplant was T cell depleted, underlining the importance of T cells as effector cells in GvL [75]. In addition, it was shown that increased natural killer T cells in the graft are associated with reduced GvHD incidence [76], whereas depletion of Tregs in DLI improves the GvL effect but on the other hand augments the risk of GvHD [77]. Thus, prophylactic and dose-escalated DLI was integrated in reduced intensity conditioning (RIC) protocols to reinforce the GvL effect and prevent disease relapse, however the risk of inducing GvHD remains [78].

The sensitivity of the underlying diseases to a DLI-mediated GvL effect is an additional factor. Response to DLI and DLI sensitivity was estimated by the relapse workshop of the National Cancer Institute [11]. CML, myelofibrosis and low-grade NHL were classified to be highly sensitive to DLI; AML, MDS, multiple myeloma and Hodgkin’s disease intermediately; and ALL and DLBCL only as slightly sensitive. Additionally, freshly infused DLI may have a higher potency compared to frozen DLI depending on different viabilities and compositions [11,56,79].

Similarly, Gröger et al. observed long-term efficacy of prophylactic donor lymphocyte infusion in 61 patients with multiple myeloma [80]. Prophylactic DLI used in escalated doses in a selected cohort resulted in a low rate of grade II–IV GvHD and encouraging long-term results in these myeloma patients. These data support the relevance of a graft-versus-myeloma effect in long-term responders after allogeneic stem cell transplantation.

## 4. Antigen-Directed Immunogenic DLI

Already in 1999, Falkenburg et al. published the idea that relapse of CML in chronic phase after allo-SCT can be successfully treated by DLI [81]. Leukemia-reactive T cell lines that could effectively elicit an antileukemic response in vivo were selected and expanded in vitro. These T-lymphocyte (CTL) lines were generated from an HLA-identical donor. Three CTL lines were generated that were able to lyse the patient leukemic cells and inhibit the growth of leukemic progenitor cells. Intriguingly, these CTL did not react with lymphocytes from donor or recipient and did not affect donor hematopoietic progenitor cells. The three leukemia-reactive CTL lines were infused at 5 week intervals at a cumulative dose of 3.2 × 10^9^ CTL. Complete eradication of the leukemic cells was observed shortly after the third infusion. The results showed that in vitro-cultured leukemia-reactive CTL lines selected for their ability to inhibit the proliferation of leukemic progenitor cells in vitro can be successfully applied to treat accelerated phase CML after allo-SCT. Based on this study further developments in AML patients after allo-SCT were possible.

Employing leukemia-specific enriched DLI could be another approach to improve the efficacy of DLI against leukemic cells, thus using immunogenic DLI directed against LAAs. These approaches may be used prospectively in selected patients to enforce GvL without inducing GvHD. The challenge will be to find the best combinations and targets to maximize the GvL- and minimize the GvHD effect, as well as the ideal cytokine milieu for therapy and the ideal T cell composition. A better understanding of the mechanisms of DLI with their targets would open doors to increase the GvL potency without raising the risk of GvHD at the same time.

One option to obtain LAA-specific T cells is the use of selection methods such as multimer approaches. Wang et al. reported about CD8+ T cells purified by streptamer technology [82]. The focus was on the immunogenic leukemia antigen WT1, the streptamer technology was employed and a 60-fold increase in WT1-specific CD8+ effector T cells after positive selection by magnetic cell separation was found. Thus, the streptamer technology allows selection of pure and antigen-specific effector T cells. These results further suggest that the functional status of CD8+ T cells purified by the streptamer technology is preserved and most purified cells are effector T cells. Therefore, these purified effector T cells could be suitable to provide immediate immune protection and might be useful for adoptive T cell transfer. However, the amount of LAA-specific T cells is low and therefore strategies for the ex vivo expansion of LAA-specific T cells have to be established. Bae et al. reported such an expansion strategy for BCMA-specific T cells in myeloma patients [83].

T cell receptor (TCR)-engineered T cells constitute another method among antigen-directed T cell approaches. Several clinical studies have been performed or are ongoing, targeting LAA like, e.g., WT-1 or PRAME. Tawara et al., demonstrated that WT-1 specific TCR-T cells manipulated ex vivo survived in vivo and induced immune responses in WT-1-positive HLA-A*24:02 positive AML and MDS patients. Furthermore, moderate clinical effects such as a decrease in blast counts in blood and bone marrow have been reported [84].

Combination strategies for these antigen-directed immunotherapeutic approaches with other immunotherapies such as immune checkpoint inhibitors might enhance or multiplicate the immune effects and are effective to eliminate leukemic cells and leukemic progenitor or even stem cells.

## 5. Specifically Stimulated and Modified DLI

### 5.1. Virus-Specific Donor T Cells for Cytomegalovirus (CMV)

CMV disease constitutes a serious complication after allo-SCT. Despite improved antiviral drug therapy used for the prophylaxis and/or treatment of CMV reactivation and disease, reactivation of CMV after allo-SCT occurs in more than 60% of CMV-seropositive patients. CMV reactivation remains a major cause for mortality and morbidity. Moreover, prolonged antiviral therapy can cause pronounced side effects, particularly myelosuppression and nephrotoxicity [85,86]. A novel prophylactic drug called letermovir showed a decrease in clinically significant CMV infection in a placebo-controlled randomized trial. Nevertheless, this prophylaxis is expensive and breakthrough infections, drug resistance as well as intolerance are still an issue [86]. Beyond humoral immune response, cell-mediated immune response is essential for the control of CMV infection and disease [87,88,89,90]. Studies demonstrated that patients are protected against CMV disease once a detectable T cell response against CMV has been mounted [91]. For prevention and therapy of CMV disease, the adoptive transfer of unmanipulated and virus-specific T cells has been evaluated in several clinical trials. [92,93,94,95]. The CMV-specific T cells are mostly derived from the donor, a third-party donor or even the patient himself prior to conditioning therapy. This specific treatment leads to virus clearance in patients after allo-SCT.

However, long-term in vitro culturing to select CMV-specific T cells is difficult and time consuming, therefore new strategies were necessary. For example, the cytokine capture assay is combined with the Miltenyi Clini MACS system to generate CMV-specific T cells. Accordingly, it was concluded that adoptive T cell therapy is a valid therapeutic option, which allowed patients to discontinue toxic antiviral drug therapy without further high-level reactivation of CMV.

An aggravation of GvHD was not observed. However, high-dose (>2 mg/kg body weight) corticosteroids could reduce the efficacy significantly.

### 5.2. Virus-Specific Donor T Cells for Epstein-Barr Virus (EBV)

EBV is widespread in all human populations and persists as a lifelong, asymptomatic infection.

Post transplantation lymphoproliferative disease (PTLD) associated with EBV is a life-threatening complication after allo-SCT [96]. In the past, the mortality from PTLD after allo-SCT was >80% [97]. Chemotherapy seems not to contribute to improved survival of patients with PTLD after allo-SCT and antiviral agents are not active against PTLD [97].

Fortunately, it was shown that by use of rituximab and the adoptive T cell transfer of EBV-specific T cells in high-risk patients, PTLD could be prevented [97], whereas EBV-specific T cells (in vitro generated donor derived or even third-party T cells) are administered in cases with EBV-DNA-emia in order to prevent EBV disease. If no response is achieved unselected DLI from EBV-positive donors are used in order to restore broad T cell reactivity including EBV-specific response (preemptive therapy 94–100% response; therapy of PTLD 71–75% response).

Another option is the treatment of established EBV-PTLD with EBV-specific T cells from the donor but there is the risk of a rapidly growing high-grade lymphoid tumor. In late-stage disease with multiorgan dysfunction at the time of T cell transfer, the results are poor [96]. In this case, the T cell therapy should be implemented as soon as possible. The main obstruction for the use of this approach is the limited availability of T cells and the urgency. To improve the availability of EBV-specific T cells in such urgent clinical situations, Moosmann et al. developed a rapid protocol for the isolation by overnight stimulation of donor blood cells with peptides derived from 11 EBV antigens, interferon-gamma surface capture and subsequent immunomagnetic separation. Therefore, protective EBV-specific T cell memory could be achieved after the infusion of a small number of EBV-specific T cells [96].

Another approach is the administration of Tabelecleucel (formerly known as ATA129) in patients with rituximab-refractory EBV-PTLD. Tabelecleucel is Atara’s off-the-shelf T cell immunotherapy in development for the treatment of EBV-PTLD, as well as other EBV associated hematologic and solid tumors. To evaluate the efficacy, a global, multicenter, open-label phase 3 clinical study, called MATCH was designed. The recruitment for MATCH (NCT03392142) is ongoing until November 2020.

### 5.3. Third Party DLI

Tzannou et al. [98] state the improvement of overall survival for patients treated with allo-SCT will require efforts to decrease treatment-related mortality caused by severe viral infections. Broad antiviral protection to recipients of allo-SCT could be provided by adoptively transferred virus-specific T cells generated from eligible, third-party donors. In their study, third-party virus-specific T cells were administered to recipients of allo-SCT with drug-refractory infections. Infusions were safe and virus-specific T cell tracking by epitope profiling revealed persistence of functional virus-specific T cells of third-party origin for up to 12 weeks. In turn, Muranski et al. developed multi-virus-specific T cells not as therapeutic, but as prophylactic approach early after transplantation [99]. In a phase I study (NIH 14-H-0182) multi virus-specific T cells (MVSTs) with the target of immunodominant viral proteins such as CMV, EBV, human polyomavirus and adenovirus were administrated immediately (day +0 to +60) after allo-SCT. Elutriated lymphocytes from sibling donors were stimulated for 14 days with seven overlapping peptide libraries (pepmixes) pulsed onto autologous DCs in presence of IL-7, IL-15 and IL-2. Twelve patients were treated. There were no infusion toxicities detected, while minimal risk of aGvHD was observed, there was no correlation found with GvHD biomarkers. By serial CDR3 sequencing, it was shown that MVSTs contribute to the T cell repertoire. This approach suggests efficacy in reducing viral reactivation. A phase II study is warranted [99]. Further studies are warranted to establish third-party antiviral T cells for clinical use.

## 6. In Vivo and Ex Vivo Manipulation of DLI for the Reduction of Alloreactive T Cells

### 6.1. In Vivo T Cell Depletion

Donor T cells are not DLI in the common sense but are very important in the field of allo-SCT. Concerning in vivo T cell manipulation, there are different approaches, for example alemtuzumab, administered intravenously or administered during the transplantation itself as “campath-1H in the bag” [100,101] as well as antithymocyte globulin (ATG) [102]. In the field of in vivo T cell depletion, the haploidentical setting is the most interesting setting [103]. Because of post-transplant cyclophosphamide (PT-Cy) [104], further advances in graft cell processing and manipulation, as well as GvHD prophylaxis, haploidentical allo-SCT is a save option for nearly all patients with AML, since a significant reduction of treatment-related mortality is now possible. However, there are limited data with respect to DLI in the setting of haploidentical allo-SCT or considering early DLI with concurrent immunosuppression [38].

### 6.2. Ex Vivo T Cell Depletion

Alpha/beta T cells are the main cell population responsible for the success or failure of allo-SCT or DLI. Expression of the alpha/beta (α/β) TCR characterizes most mature T cells, which allows MHC-restricted recognition of peptides derived from non-self-proteins. The T cell repertoire after allo-SCT is influenced by the source of the graft and infectious challenges such as CMV and EBV, as well as GvHD and cellular intervention such as DLI. Depletion of naïve T cells from the graft is a promising approach to prevent GvHD while retaining a strong GvL effect [32]. Attempts are encouraging on the one hand against hematological tumor antigens for the treatment of overt leukemia relapse and on the other hand to enable a faster immunreconstitution after allo-SCT [105]. The repertoire of α/β T cells after allo-SCT has been studied in different allo-SCT settings and is still restricted 6 months after allo-SCT when compared to healthy individuals. Surprisingly cord blood grafts lead to a higher diversity of the α/β TCR repertoire at 6 and 12 months compared to other graft sources [106].

It has been shown that the main cell population responsible for the success or failure of allo-SCT or DLI is α/β T cells [53]. Since most alloreactive α/β T cells are present in the naïve repertoire of the donor, recipient-derived DCs are key players in producing an appropriate T cell activation [107]. DCs are derived from the hematopoietic system and therefore generate a recipient targeting immune response, including the malignant population, and therefore give rise to GvL [107]. The level of cross reactivity against antigens expressed on non-hematopoietic cells determines the likelihood and severity of GvHD.

In T cell-repleted allo-SCT, it is difficult to dissect the GvL and GvHD effect [108,109]. Consequently, many current transplantation techniques remove immune cells from the graft and administer DLI at a later time point as standard part of the transplantation regimen. Both a complete immune depletion by selection of CD34+ stem cells [110], and a partial depletion of alloreactive T cells through PT-Cy [111], are feasible. This upfront T cell depletion is associated with a lower risk of GvHD and allows early DLI administration for the majority of patients (e.g., 100 days) after allo-SCT. An improved segregation of the GvL- and GvHD effect is possible due to this approach. More recent transplantation strategies allow better consideration of the sophisticated variety of immune cells. These novel strategies utilize either a selective depletion of α/β T cells [112] or naïve subsets [32] to abrogate GvHD, while maintaining early immune surveillance directed against infections as well as leukemia.

One strategy to eliminate alloreactive T cells and at the same time protect virus-specific memory T cells is the ATIR101 program. is a new approach to reduce risk of GvHD after allo-SCT. It consists of a single DLI dose with functional, mature immune cells from a haploidentical family member. Thus, protective T cells are preserved to fight relapse and infections and reduce risk of GvHD. Alloreactive T cells are depleted ex vivo. So far, the results are promising; hence, this approach may increase the safety of allo-SCT from a haploidentical family donor.

## 7. Five-Year View

The T cell repertoire after allo-SCT is not as diverse as in healthy individuals [106,113]. GvHD is associated with both an increased [106] and a decreased diversity [114]. Selective GvL reactivity could be associated with lower diversity, lower magnitude and relatively specific tissue recognition of hematopoiesis by alloreactive α/β T cells [115]. The contribution of the diversity of the γ/δ TCR repertoire to the GvL after allo-SCT is not well described. The γ/δ T cell repertoire seems to be established quite early after 30–60 days after allo-SCT. CMV reactivation promotes the massive expansion of a few γ/δ T cell subtypes (belonging mainly to the delta-1 subset) resulting in a so-called repertoire focusing [116]. Henceforth, DLI administration for prophylactic, preemptive and overt relapse, as well as treatment of prophylaxis of infections, or immune reconstitution might not only depend on the type of the disease, or the timing but also on the size of the α/β and γ/δ T cell repertoire monitored at a given time point.

## 8. Conclusions

DLI after allo-SCT offers great opportunities with regard to the treatment or prophylaxis of relapse as well as preemptive treatment of a persistent MRD or decreased chimerism in AML/MDS patients. Furthermore, DLI may be administrated in the setting of treatment or prophylaxis of viral infections and provide substantial support with regard to immunreconstitution.

However, there are many aspects involved in the application of DLI. DLI application is a difficult intervention, whereby many factors have to be considered, for example individual patient-oriented factors prior to application, as well as possible combinations with further therapies during and after DLI application. Currently, there are many new developments, and this is quite necessary because DLI needs to be improved in terms of efficacy and toxicity reduction. This remains a major challenge with the goal to improve the outcome for AML patients after allo-SCT. Prospectively, *CAR* T cells may be an intriguing concept even in AML, with the possibility of leukemia rejection without eliminating healthy progenitor and stem cells. However, there is no feasible approach yet.

In this field, of manipulated and unmanipulated DLI after allo-SCT of AML patients, it remains most challenging to avoid substantial risks such as severe infections and several key points, such as dosage, donor origin, as well as the clinical situation of the patient, have to be considered prior to DLI administration. The pivotal point is to increase the GvL effect without escalating the risk of GvHD.

## Figures and Tables

**Table 1:** Prophylactic, preemptive and therapeutic studies in DLI. Conditions and response rates of studies and corresponding references.

Situation	Patients, Study Type	Strategy	Response	Reference
prophylactic	retrospective, matched n = 46, high-risk AML/MDS	DLI, +120 d post allo-SCT	7-yr OS, 67% vs 31% (p < 0.001) OS in the DLI group was significantly improved	Jedlickova et al. BMT, 2016
	retrospective, high-risk AML	DLI, post allo-SCT	OS in DLI group was improved 70% vs. 40% (p = 0.027)	Schmid et al. Br J Haematol, 2019
preemptive	n = 105, prospective standard-risk AML, ALL, MDS	49 low-dose IL-2 56 DLI	3-yr OS: DLI: 58%, IL-2: 28%	Yan et al. Blood, 2012
	n = 101, MDS/AML	preemptive chemotherapy in combination with DLI application	CIR, NRM and DFS 39.5%, 9.6%, and 51.7%	MO et al. Eur J Haematol, 2016
	prospective phase II study n = 20, MDS/ AML	> 100 d post allo-SCT four azacytidine cycles (75 mg/m^2^/d for 7 days)	hematological relapse in 13 patients (65%)	Platzbecker et al. Leukemia, 2012
therapeutic	retrospective, 399 patients	177 DLI, 228 no DLI	2 yr OS 21% with DLI, 9% without DLI (p = 0.04)	Schmid et al. J Clin Oncol, 2007
	n = 263, retrospective	cytoreductive therapy, followed by DLI or second HSCT	CR was reinduced in 32%; 2 yr OS was 14%	Schmid et al. Blood, 2012
	n = 57, prospective	cytarabine-based therapy and DLI	2 yr OS 19%	Levine et al. J Clin Oncol, 2002
	prospective phase I study AML	azacytidine post DLI	CR (6/8)	Ghobadi et al. Leuk Res, 2016
	retrospective, MDS/AML	azacytidine/DLI	Overall response was 33% OS after 2 yrs 29%	Schroeder et al. BBMT, 2015
	retrospective, AML	Sorafenib, in combination with hypomethylating agents and DLI	38% CR	De Freitas et al. Eur J Haematol, 2016

Legend: CIR=cumulative incidence of relapse; CR=complete remission; d=days; DLI=donor lymphocyte infusion; IL-2=interleukin-2; n=number; NRM=non-relapse mortality; OS=overall survival; yr=year.
